# Rare manifestations of cutaneous diphtheria: a case report

**DOI:** 10.1186/s13256-024-04646-5

**Published:** 2024-09-06

**Authors:** Salma Ali Suwaid, Aliyu Mustapha, Monika Ried, Ruqayyah Ali Muhammad, Aminu Shehu Liman, Salma Yusuf

**Affiliations:** 1 Department of Pediatrics, Diphtheria Treatment Center, Murtala Muhammad Specialist Hospital (MMSH), Kano, Nigeria; 2 Hospital Management Board (HMB), Kano, Nigeria; 3 Vanke School of Public Health, Tsinghua University, Beijing, China; 4 Ahmadu Bello University, Zaria, Nigeria; 5 Klinikum München Bogenhausen, Munich, Germany

**Keywords:** Diphtheria, Cutaneous diphtheria, Pseudomembrane, Sitz baths, Hydrogen peroxide, Psychosocial context

## Abstract

**Background:**

This case report presents an exceptionally rare and atypical presentation of diphtheria in a 17-year-old female of Hausa ethnicity residing in an area with an elevated incidence within Kano State, Nigeria. By the end of 39th epidemiological week of 2023, only two cases of cutaneous diphtheria have been reported among 5,811 cases managed at MMSH diphtheria treatment center (DTC), representing approximately 0.035% of all diphtheria cases during that time period.

**Case presentation:**

A 17-year-old Hausa female presented with a 3-day history of throat discomfort, malaise, and muffled speech. Physical examination revealed a pseudomembrane covering the tonsillar pillars, grade 3 tonsillar enlargement, and an unusual genital manifestation characterized by extensive vulval edema, severe pain, and a large, greyish patch extending into the vaginal introitus. Her medical history was significant for recent exposure to diphtheria and the emotional impact of her sibling's death from the same disease. Diagnostic tests, including throat swab culture and histocytology, confirmed diphtheria in the throat and vulvovaginal regions. The patient was promptly initiated on diphtheria antitoxin, Azithromycin, and innovative sitz baths with hydrogen peroxide. After 4 days of Sitz bath therapy, complete pseudomembrane clearance was observed, and the patient's symptoms resolved.

**Conclusion:**

This case underscores the complexity of diphtheria presentations, particularly with rare pseudomembranes in both throat and vaginal regions. The successful management, combining traditional and innovative therapies, highlights the importance of recognizing and addressing unusual manifestations promptly. The potential role of auto-inoculation and the efficacy of interventions like hydrogen peroxide sitz baths warrant further investigation. Overall, this case contributes to the understanding of diverse diphtheria presentations, guiding future clinical strategies for management of diphtheria cases and emphasizing the imperative of comprehensive vaccination efforts.

## Introduction

Cutaneous diphtheria, a rarely seen manifestation of Corynebacterium diphtheriae, presents a unique challenge in clinical practice, particularly in regions with high prevalence of poverty [1]. Despite its infrequency, cutaneous diphtheria has raised concerns due to its association with distinct risk factors, including poor socioeconomic status [1]. Cases of cutaneous diphtheria carry significant implications, as transmission can occur through contact with respiratory secretions, infected skin lesions, or exposure to contaminated dust and fomites [2]. Interestingly, the transmissibility of cutaneous diphtheria is suggested to exceed that of its respiratory counterpart [3], highlighting its potential threat to public health by fostering the potential for localized outbreaks, especially in densely populated areas with insufficient vaccination coverage, a single case could trigger a chain of infections, leading to clusters of disease[4]. This not only strains healthcare resources but also elevates the risk of further transmission within the communities.

Comprehensive data on cutaneous diphtheria remain scarce, necessitating an in-depth exploration of its clinical dynamics, especially in the context of an ongoing outbreak. This study focuses on an intriguing subset within the diphtheria outbreak in Kano State, Northern Nigeria [5], where the MMSH Diphtheria Treatment Center recorded a notable 5811 diphtheria cases between January and September of this year, pinpointed to Epi-week 39. Among these, we characterize two cases of cutaneous diphtheria identified and managed at the center, with one case standing out as a poignant illustration of the disease's complexity.

This study places a particular emphasis on the evolution of a seventeen-year-old patient with a remarkable manifestation of cutaneous diphtheria, presenting as a lesion localized in the vaginal region. Notably, this case also featured an intriguing co-occurrence of respiratory diphtheria. We document the clinical journey of this patient, capturing the nuanced changes that occurred throughout the course of treatment.

## Case presentation

A 17-year-old Hausa female student from Giginyu, Nassarawa Local Government Area (LGA), Kano State, presented with fever and difficulty swallowing. She lives with her parents and siblings in a region with a high incidence of diphtheria. The patient was not vaccinated against diphtheria. She has no significant past medical history other than minor ailments like common colds and malaria, which were managed at home. Her menstrual cycles are regular with no abnormal gynecological history, and she has no history of smoking or alcohol consumption and she is not sexually active.

On admission, her clinical examination revealed a throat pseudomembrane and grade 3 tonsillar enlargement. Her vital signs were as follows: blood pressure was 110/70 mmHg, oxygen saturation (SpO2) of 99% on room air, and pulse rate of 120 bpm, regular and full volume. She was conscious and oriented to time and place throughout her hospital stay, with no neurological abnormalities noted.

Laboratory findings were as follows: CBC showed a red blood cell count (RBC) of 4.83 × 10^9/L, white blood cell count (WBC) of 9.5 × 10^9/L, and platelet count (PLT) of 332 × 10^9/L. Biochemistry results included urea at 3.3 mmol/L, creatinine at 62 umol/L, sodium at 145 mmol/L, potassium at 3.88 mmol/L, bicarbonate (HCO3) at 28.9 mmol/L, and chloride at 108 mmol/L. No urinalysis was conducted, and no radiographic scans were performed due to the absence of indications for such diagnostics.

During her hospitalization, the patient was treated with 60,000 International Units of diphtheria antitoxin (DAT), administered intravenously in 250 ml of normal saline over 4 h, intravenous dexamethasone (24 mg initially, followed by 4 mg every 6 h for 3 days), intravenous paracetamol (1 g every 8 h for 4 days), oral azithromycin (500 mg daily for 14 days), and oral gargles with warm saline water three to four times daily, along with other supportive care.

### Clinical progression and innovative treatment

**Fig. 1:**
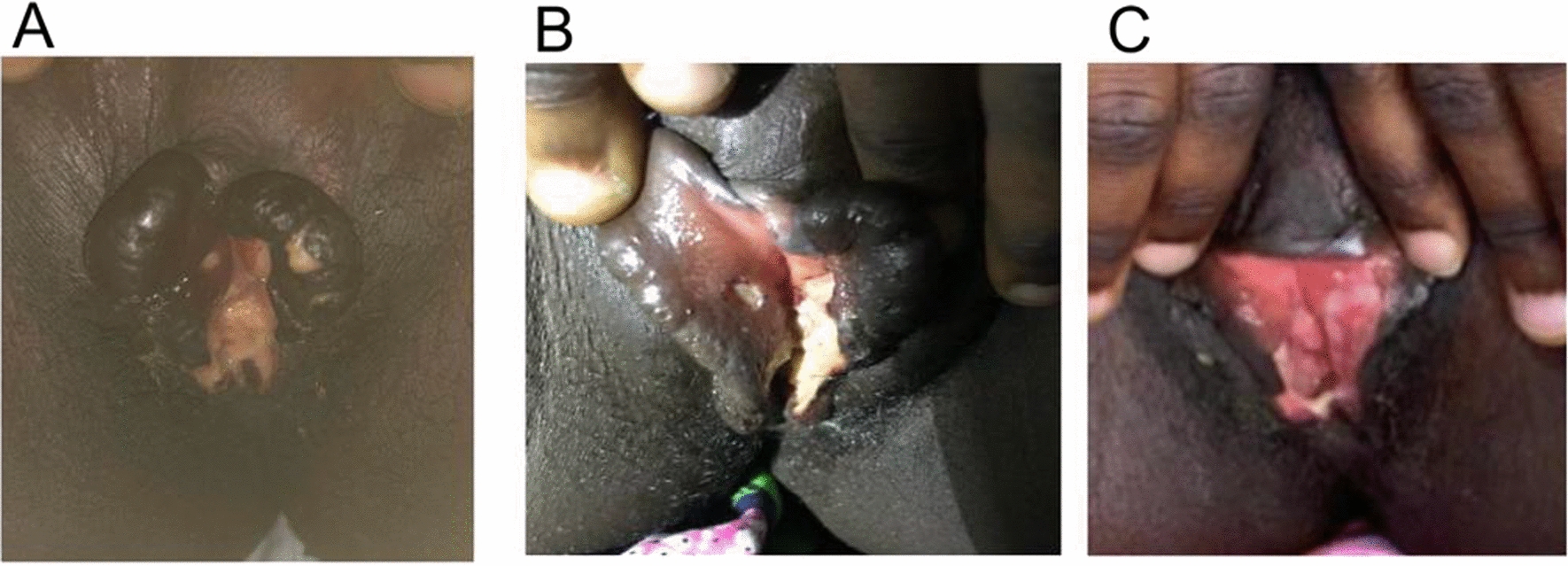
**A** Greyish-white patch on day 4 of admission **B** After 3 days of sitz bath with hydrogen peroxide **C** After 8 days of management in DTC

Four days following admission, despite stable blood parameters (CBC, creatinine, urea, and electrolytes), the patient reported new symptoms of vulval swelling and pain. Clinical examination revealed an edematous vulva with a greyish-white patch extending into the vaginal introitus (Fig. 1A). Positive culture and histocytology results confirmed diphtheria involvement in these regions, marking an atypical manifestation of the infection. Given the unusual site and presentation, an innovative treatment approach was initiated. The patient underwent sitz baths with a 1:10 dilution of 6% hydrogen peroxide administered twice daily. This treatment was selected based on its oxidizing properties, hypothesized to aid in the disruption of bacterial biofilms and toxin inactivation. Remarkable improvement was observed within four days, with complete clearance of the pseudomembrane documented (Fig. 1B).

Following an eight-day hospitalization period, the patient was discharged with all symptoms resolved (Fig. 1C) and was prescribed oral azithromycin, 500 mg daily, for an additional six days. A follow-up visit two weeks later confirmed sustained improvement, with no recurrence of symptoms. Additionally, the patient received the diphtheria vaccine upon the second follow-up three weeks post-admission, aiming to enhance individual immunity and contribute to community protection.

## Discussion

The presented case adds to the understanding of cutaneous diphtheria, a manifestation typically associated with toxigenic or non-toxigenic strains of *C. diphtheriae*. Cutaneous diphtheria is generally considered a milder form of the disease, leading to cutaneous sores or shallow ulcers. It is noteworthy that toxic complications in cutaneous disease are rare [6, 7]. The case characterized the simultaneous presence of pseudomembranes in both the throat and vaginal regions. While it remains unclear whether these presentations were attributable to the same strain of *C. diphtheriae* or distinct strains, the co-occurrence of these manifestations raises intriguing questions about the mode of transmission and pathophysiology involved.

The rarity of this dual presentation prompts speculation that the infection might have resulted from auto-inoculation, whereby the organism was transferred from one part of the body to another by the patient. Auto-inoculation could have occurred through inadvertent contact or transfer of contaminated secretions. Additionally, while the patient reported that she is not sexually active, it is important to consider and rule out all potential modes of transmission, including the theoretical possibility of transmission through oral sex with an infected person. The lack of genetic characterization impedes our ability to definitively ascertain the origins and relatedness of the strains involved. Further investigation through molecular typing and genomic sequencing could offer valuable insights into the potential sources of infection and shed light on the mechanisms underlying these unusual dual manifestations [8].

Remarkably, the implementation of a diluted hydrogen peroxide sitz bath emerged as a successful intervention in our center. This innovative approach yielded significant improvements, leading to the complete clearance of the vaginal pseudomembrane. While the exact mechanism of action remains speculative, studies on the effectiveness and safety of hydrogen peroxide solutions in treating different conditions, such as genital warts [9] and non-genital warts [10], highlight its potential as a therapeutic option. However, it's crucial to recognize the distinctions between these conditions and emphasize the necessity for specific research on hydrogen peroxide's efficacy in treating diphtheria manifestations. Further investigation is warranted to elucidate the benefits, limitations, and reproducibility of this treatment strategy across diverse patient populations and clinical settings.

The patient's lack of immunization against diphtheria is a notable contributing factor in this case, emphasizing the significance of vaccination coverage. In the broader context, the low vaccination coverage in Nigeria, with only 57% of children receiving three doses of the vaccine in 2022 [11], highlights the challenges in achieving comprehensive immunity. This deficiency likely predisposed the patient to the rare presentation of cutaneous diphtheria, further underscoring the urgent need for enhanced vaccination programs. This case serves as a reminder of the ongoing relevance of vaccination campaigns, especially in regions with low coverage, to effectively reduce the incidence of diphtheria and its potential complications.

Furthermore, investigation into the healing timeliness of cutaneous diphtheria unveils intriguing variations in the response to treatment. A comparison with fully vaccinated child from previous study, who exhibited persistent pruritic lesions persisting for over 2 weeks, ultimately requiring a change in antibiotic therapy to achieve healing within the following week [12]. In contrast, the non-vaccinated child in our case exhibited a remarkably rapid resolution of symptoms, with complete healing observed in less than a week. This discrepancy underscores the complexity of cutaneous diphtheria's clinical course and prompts further exploration into potential contributing factors such as vaccination status and strain variability.

## Conclusion

This case report highlights the effectiveness of combining traditional diphtheria treatments with hydrogen peroxide sitz baths, which facilitated rapid symptom resolution in a patient with unusual pseudomembrane formations in the throat and vaginal areas. Such presentations emphasize the necessity for clinicians to be prepared for diverse manifestations of diphtheria. The patient's severe condition, linked to incomplete vaccination, reinforces the essential role of thorough vaccination programs. This case serves as a stark reminder of the consequences of inadequate immunization and the need to enhance vaccination efforts.

Further investigation is needed to assess the safety and effectiveness of hydrogen peroxide sitz baths and other novel treatments for diphtheria. Future research should focus on validating these innovative approaches to broaden therapeutic options for infectious diseases.

## Data Availability

The data supporting the findings of this study are included within the article. Any additional data or materials related to this study are available from the corresponding author upon reasonable request.

## Availability of data and materials

All data generated during this study are included in this published article and its supplementary information files.
